# Physicochemical Characteristics of Potato Starch Extrudates Enriched with Edible Oils

**DOI:** 10.3390/molecules31020293

**Published:** 2026-01-14

**Authors:** Marzena Włodarczyk-Stasiak, Małgorzata Jurak, Agnieszka Ewa Wiącek

**Affiliations:** 1 Department of Analysis and Evaluation of Food Quality, Faculty of Food Science and Biotechnology, University of Life Sciences in Lublin, Skromna Street 8, 20-704 Lublin, Poland; 2 Department of Interfacial Phenomena, Institute of Chemical Sciences, Faculty of Chemistry, Maria Curie-Skłodowska University in Lublin, Maria Curie-Skłodowska Sq. 3, 20-031 Lublin, Poland; malgorzata.jurak@mail.umcs.pl

**Keywords:** potato starch extrudate, K_2_CO_3_ catalyst, edible oils, FTIR, static/dynamic light scattering, electrophoresis

## Abstract

Starch systems and their extrudates can be used as edible films, carriers, and encapsulants for bioactive substances in various industries, primarily the food, medicine, and pharmacy industries. Using appropriate modification methods, it is possible to alter their physicochemical properties to improve specific functional parameters, thereby enhancing their application potential. The aim of this study was to characterize potato starch extrudates enriched with two types of edible oils (rapeseed or sunflower) at concentrations of 3%, 6%, and 9%. Chemical modification was carried out using K_2_CO_3_ as a catalyst. The structure of native and modified starch extrudates was examined using optical/confocal microscopy, FTIR, and LTNA (low-temperature nitrogen adsorption). Analogous starch dispersions were studied using static and dynamic light scattering, SLS/DLS, nephelometric methods, and electrophoretic mobility measurements to determine surface charge levels and stability. Additionally, viscosity curves were determined as a function of time and temperature. It was found that starch extrudates with 6% sunflower oil content showed optimal functional properties, characterized by greater stability, higher structural order, and better oil complexation. These findings directly translate into significant potential applications, including the development of functional products in the food industry.

## 1. Introduction

Starch is a polysaccharide composed of glucose building blocks, which form linear amylose chains via α(1,4) glycosidic bonds and branched amylopectin chains via α(1,4) and α(1,6) glycosidic bonds. Starch is a common plant material, readily available in pure form, making it inexpensive and thus increasing its applications. The botanical origin of starch determines the proportion of these types of polymers in the molecule and the size of its granules. However, certain physicochemical properties of starch, such as high polarity, low solubility in organic solvents, and hydrophilicity resulting from the presence of three free hydroxyl groups in each anhydroglucose unit [1], can limit its use in various industries [2].

Therefore, specific properties of starch can be created by physical, chemical, and/or enzymatic modifications [3,4,5]. Regardless of the method, each of them causes changes in the composition or structure of the starch [3]. Due to the relatively high potential for developing novel starch properties, two or more different types of modifications are often combined in so-called mixed methods. The most popular methods include physical modifications, which generally involve the action of one or more physical factors on starch, such as temperature, pressure, hydrothermal treatment, mechanical force, irradiation, or ultrasound. A significant advantage of these methods is the relatively broad range of physical factors and the absence of reagents supporting modification (e.g., chemical) in production, which is of great importance when modifying starch used in the food industry. An interesting approach to modifying the properties of starch is extrusion, which combines factors such as temperature, pressure, mixing, and hydrothermal action. Depending on the selection of raw materials and process parameters, the final product is an extrudate ranging from a highly developed porous surface to a compact structure or film.

Another equally popular group is the chemical modification of starch, typically achieved through esterification, etherification, or oxidation. These approaches lead to starches with properties different from native starch and varying degrees of substitution (DS), often cross-linked. Among these, esterification is the most prevalent method. Its general mechanism involves the substitution of hydroxyl groups of the starch molecule with an esterifying compound, usually in the presence of a catalyst. The efficiency of esterification depends on the availability and reactivity of the three hydroxyl groups within each anhydroglucose unit. A measurable indicator of process efficiency is the degree of substitution (DS), reaching the maximum value of 3.

A quite promising modification described in the literature is the esterification of starches of various botanical origins [6,7] with short-chain carboxylic acids [8,9] or long-chain fatty acids [2,9,10,11]. The hydrophobic character of esterified starches varies depending on the length of the fatty acid chains and the degree of substitution [3,12], and increases with increasing DS. Furthermore, starches esterified with fatty acids exhibit the characteristics of thermoplastic materials, and their biodegradability is enhanced [3,11,13,14,15].

Such starches can be a valuable raw material in the food industry, particularly for food-grade films or even edible coatings. Besides the obvious benefits of food protection, starch-based packaging reduces the costs of producing additional packaging, the risk of hazardous ingredients migrating from packaging into food, and finally, the environmental burden of packaging waste.

However, despite the obvious advantages of enriching starch with edible oils, there are also challenges. Long-chain fatty acids used for esterification can contribute to limited substitution (low DS) due to steric hindrance [10]. Therefore, selecting the optimal fatty acid can be challenging. The original hydrophilicity of starch, resulting from the presence of numerous hydroxyl groups in the molecule, and intermolecular and intramolecular hydrogen bonds due to substitution with hydrophobic reagents, leads to the formation of a specific hydro/lipophilic state [3] in the starch ester. Such a promising dual character (hydrophilic/lipophilic) of esterified starches prompted in-depth research on combining chemical modification (esterification) with physical modification (extrusion). Commercial potato starch was extruded with two edible oils (rapeseed and sunflower) in the presence of a catalyst. To ensure relevance for food industry applications, these specific oils were selected because they are well-researched, readily available, and generally accepted food oils. As characterization of specific properties of these systems in our previous studies [5] yielded promising results, we decided to continue our research.

The potential for use in food products and growing consumer awareness (clean label trend) allows for simple information about the composition, additives used, packaging components, and edible coatings. Therefore, we sought a catalyst that would effectively facilitate reactive extrusion and starch-oil complexation while remaining a widely accepted food additive. Potassium carbonate (K_2_CO_3_) is well-documented in the literature as a buffering, pH-stabilizing, and leavening agent. It is commonly added to carbonated beverages, cheeses, and as a leavening agent in confectionery. Most importantly, it is legally compliant with food safety requirements and is designated as E501. In this study, K_2_CO_3_ was used as a catalyst to increase the efficiency of the chemical modification, following the innovative methodology described in detail in our previous papers [5,16]. Its catalytic efficiency was confirmed by a relatively high degree of substitution (~2.5) [16,17,18,19]. The novelty of this research lies in the unique composition of the mixture intended for extrusion (edible oils and a leavening agent) and, simultaneously, the use of methodology suitable for modified extrudates as standalone carriers and for the same extrudates in typical aqueous/glycerol dispersion systems, thus expanding their applicability.

The aim of the research was to obtain extruded starch modified with edible oils through a catalyst-supported physicochemical modification; to evaluate the effect of modification on the structure and hydro/lipophilicity of starch systems, porosity, adsorption, and electrokinetic properties, and potential functional properties depending on the type of oil used and as a function of its concentration. We hope that based on the conducted research, it will be possible to indicate which of the oils (rapeseed or sunflower) used provides the most effective modification of the starch system.

## 2. Results and Discussion

### 2.1. Microscopic Analysis

Optical microscopy was used to study the surfaces of basic extrudates and those enriched with two types of oils as a function of their concentration. Due to the low resolving power of classic microscopy, which limits the ability to precisely observe starch structure before and after oil modification, a more advanced methodology utilizing a confocal attachment was subsequently applied. It is well-known that the analyzed samples may exhibit the ability to naturally fluoresce or may be labelled with fluorescent dyes. These dyes, whether covalently bound or attached through other types of physicochemical interactions between substances, allow for the precise visualization of specific structures or even processes when excited with light of a defined wavelength (408 nm in this particular case).

Table 1 and Table 2 show images of the extruded starch (C) and starch extrudates enriched with rapeseed (R) or sunflower (S) oil, respectively, obtained from both optical and confocal microscopy. The effect of increasing the oil concentration in the 3–9% range is particularly noticeable in the confocal microscopy images. The control starch exhibits a typically heterogeneous system with considerable variation in both shape and size. The average size of extruded starch is in the range of average of 750–1000 µm, while after extrusion with rapeseed oil, the granules are smaller (average of approximately 250–750 µm, not counting the shrapnel) and even more variable. Differences between samples with various rapeseed oil concentrations are visible, but finding a clear correlation is difficult. In the case of the starch extrudates enriched with sunflower oil at different concentrations, significant variation in both shape and size is also observed. The granules are slightly larger (approximately 300–800 µm) than those modified with rapeseed oil, with less sharp shapes, likely due to the higher viscosity of these systems. On the other hand, these images (Table 1 and Table 2) correlate quite well with data obtained from other experimental techniques (e.g., Dynamic Light Scattering, DLS), discussed in the subsequent sections.

### 2.2. Viscosity Curves as a Function of Temperature and Time Gradient

**Figure 1:**
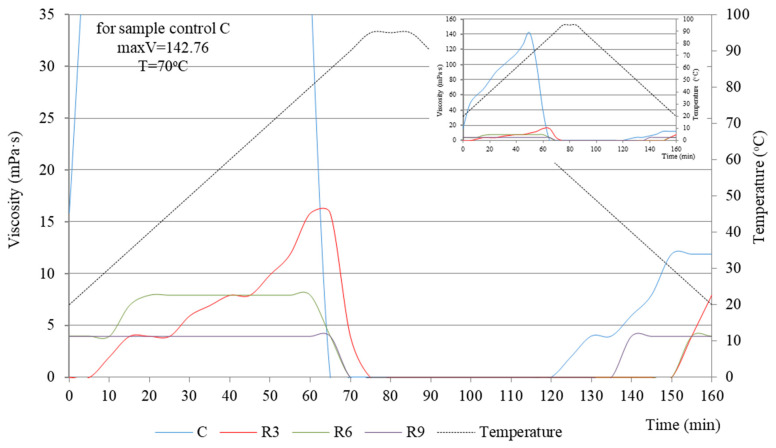
Viscosity curves vs. the temperature and time gradient of starch extrudates with and without (C) increasing rapeseed oil concentrations (3% R3, 6% R6, and 9% R9).

**Figure 2:**
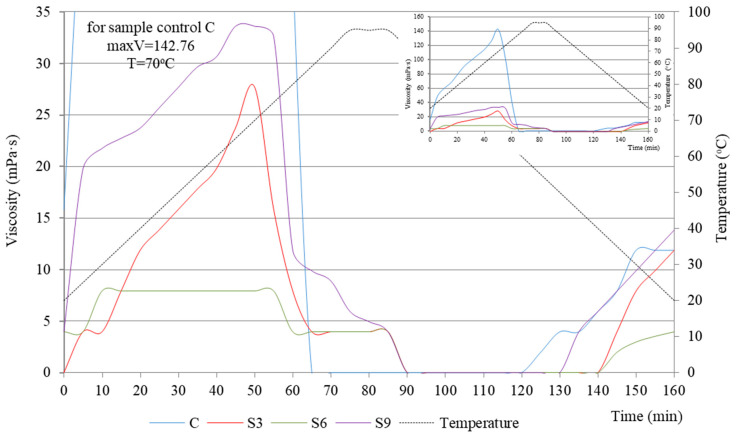
Viscosity curves vs. temperature and time gradient of starch extrudates with and without (C) increasing sunflower oil concentrations (3% S3, 6% S6, and 9% S9).

To obtain a complete physicochemical characterization, viscosity tests were conducted on the starch extrudates with and without (C) increasing oil concentrations. Figure 1 and Figure 2 present the obtained relationships as a function of time and temperature. The temperature–time gradient viscosity curves for oil-enriched extrudates and the control sample (C) exhibited distinctly different profiles. With increasing rapeseed oil content (Figure 1), the peak viscosity values decreased significantly: R3 (15.82 mPa·s) > R6 (7.91 mPa·s) > R9 (3.96 mPa·s), compared to the control sample peak of 142.76 mPa·s. During heating, after exceeding the temperature of 85 °C, all the samples reached a viscosity close to zero, which also remained unchanged during cooling of the systems to approximately 40 °C. Oil can inhibit starch–water interactions, leading to a reduction in the viscosity of the entire system. This depends strictly on the type of oil used and its structure, particularly the length of the hydrophobic chains [20,21]. This phenomenon is further supported by the FTIR results described in the subsequent sections (see Section 2.3).

On the other hand, the viscosity curves for starch systems with varying sunflower oil contents (Figure 2) do not show a similar decreasing trend observed with rapeseed oil (Figure 1). Instead, the obtained values are higher, even twofold.

For samples S9 and S3, the viscosity peak was reached at 70 °C, at 33.63 mPa·s and 27.70 mPa·s, respectively. The lowest viscosity was obtained for sample S6 (7.90 mPa·s), very close to the value for sample R6 (7.91 mPa·s). Given the practical applications of this research, the behavior of sample S6 is particularly noteworthy. This sample achieves a relatively low peak viscosity, which is maintained in the temperature range from 30 °C to 70 °C, indicating its high stability during heating. Only after exceeding 70 °C, the viscosity drops significantly by nearly 50% to 3.9 mPa·s, which persists during annealing (90 °C). Upon cooling, sample S6 reaches a viscosity close to 0 mPa·s, consistent with samples S3 and S9 (Figure 2).

Analogous viscosity measurements were performed for glycerol systems as a function of time and temperature gradients. The viscosity curves showed no significant variation during heating and cooling of the systems. Because the results for the analyzed samples and the glycerol control were identical, further discussion of these curves has been omitted.

The variations in viscosity curves over temperature and time gradients for samples enriched with vegetable oils during extrusion align with our previous findings [5]. We analyzed the functional properties of these samples, such as water solubility (WSI) and fat absorption (FAI). Correlations were noted between low WSI values (5–9%) and high FAI values (150–170%) [5]. Therefore, we can assume that the newly formed structure of the starch–edible oil extrudate exhibits a weak affinity for water molecules (low WSI) and/or the formation of structures that limit water accessibility. The ability of water to penetrate these newly formed extrudate structures is a key factor in swelling and water retention (WAI), which clearly influences the viscosity of the systems.

As was mentioned earlier, starch enrichment with vegetable oils during extrusion was carried out using a K_2_CO_3_ catalyst. This resulted in the accumulation of OH^−^ or CO_3_^2−^ anions, which likely participate in the disruption of hydrogen bonds within the starch structure [22]. This process can be considered both advantageous and disadvantageous. On one hand, CO_3_^2−^ ions bind to specific sites in the starch chain, contributing to the formation of a hydration zone, which increases the viscosity of gels [23,24]. On the other hand, excessive amounts can cause a rapid decrease in solution viscosity due to disruption at the sites of hydrogen bonds [25].

### 2.3. FTIR Analysis

**Figure 3:**
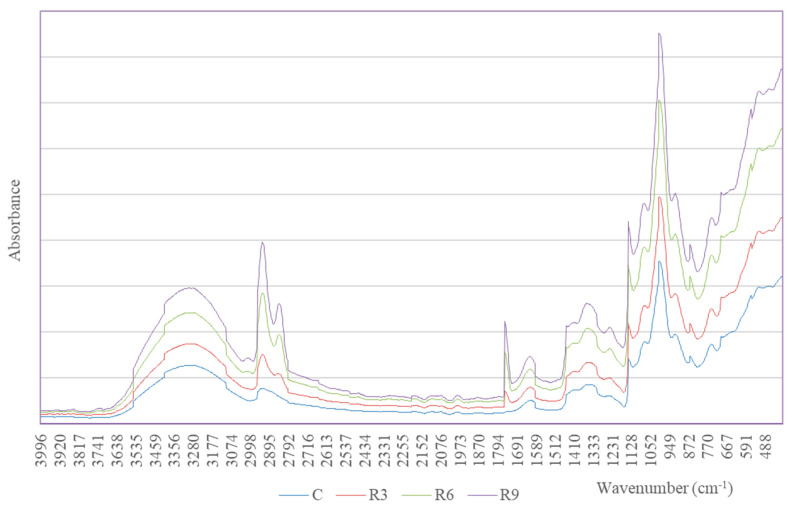
FTIR spectrum of starch extrudates with and without (C) increasing rapeseed oil concentrations (3% R3, 6% R6, and 9% R9).

**Figure 4:**
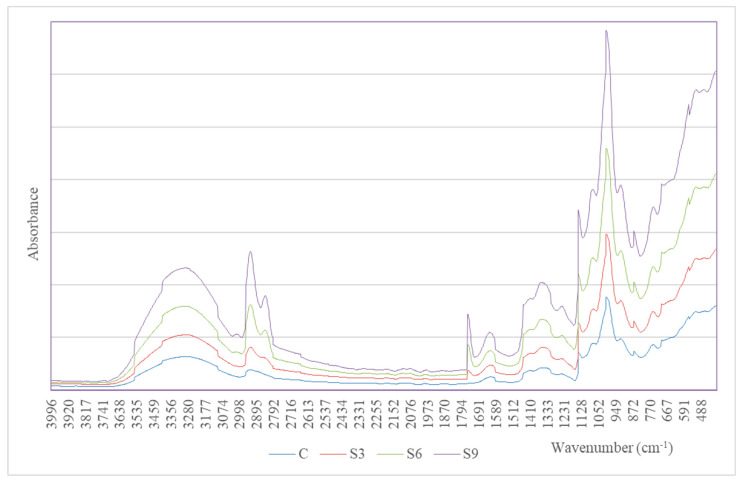
FTIR spectrum of starch extrudates with and without (C) increasing sunflower oil concentrations (3% S3, 6% S6, and 9% S9).

FTIR analysis for the starch extrudates with and without various amounts of edible oil was performed in the 400–4000 nm range. The measurement results, in the form of absorption spectra, are presented in Figure 3 and Figure 4 for the samples with rapeseed oil (R) or sunflower oil (S), respectively. These spectra allow for determining the direction of structural changes, effects of enrichment with oil, and the approximate identification of newly formed structures.

After modification of the starch extrudates, key changes were observed in the 1735–1750 cm^−1^, 1170–1250 cm^−1^, 2850–2950 cm^−1^, and 1450–1470 cm^−1^ ranges, involving the appearance of new peaks or changes in the intensity ratio of existing peaks. Specifically, the appearance of quite distinct peaks in the range of 1741–1743 cm^−1^ was observed for the extrudate samples with added oils, which were not present in the control sample, C. This peak indicates the presence of a C=O stretching group, characteristic of ester carbonyl groups [17,26]. Therefore, the formation of starch esters of food oils is possible, but confirmation requires in-depth and extended studies, which we are planning.

The intensity ratio of the mentioned peaks increases with the share of food oil in the sample (R3 and S3) < (R6 and S6) ≤ (R9 and S9). For the extrudates containing sunflower oil, the intensity ratio ranges from 0.024 to 0.050 (Figure 4), while for the samples with rapeseed oil, it ranges from 0.020 to 0.036 (Figure 3). These results suggest a stronger complexation of sunflower oil by starch, where repeating cross-linking units may contribute to the stabilization of the extrudate structure. In addition, higher intensity ratios for the samples extruded with sunflower oil (S), from 0.058 to 0.095, may indicate higher structural order, while lower intensity ratio values (0.044–0.050) for the samples with rapeseed oil (R) suggest a looser structural arrangement.

Another characteristic range is 1450–1470 cm^−1^, in which the appearance of a peak indicates the presence of CH_2_/CH_3_ groups, corresponding to the deformation of aliphatic chains in the ester structure. The presence of C–O–C groups, to which stretching -C–O bond vibrations are assigned, is confirmed by the appearance of distinct bands in the range of 1170–1250 cm^−1^ and C–O–C bands (1170–1250 cm^−1^), responsible for glycosidic bonding in polysaccharides [27,28].

In the range of 2850–2950 cm^−1^, a relatively broad band with two peaks was observed for the extrudates with added edible oils, with the intensity ratio increasing in proportion to the oil content (Figure 3 and Figure 4). This intensity ratio is due to C–H stretching groups and typical bands for –CH_2_– and –CH_3_ groups in aliphatic chains, commonly associated with fatty acid esters. Based on these intensity ratios, it can be assumed that samples S6 and S9 exhibited the strongest complexation of edible oil during extrusion (Table 3). This hypothesis is supported by previous pilot studies by Włodarczyk-Stasiak [5], which analyzed the fat complexing capabilities of these samples. On the other hand, the intensity ratio at 1047 cm^−1^/1022 cm^−1^ is considered an indicator of areas with starch crystallinity [28]. The highest value was recorded for sample R9 (intensity ratio 1047/1022—0.7543) (Table 3), suggesting the formation of a new, highly ordered structure during extrusion, which indirectly confirms the previous conclusion [28,29].

For better illustration, Table 3 presents the most important groups of bonds vs. intensity ratio and interactions for the tested samples. This comparison highlights the distinct structural differences between the individual starch extrudates.

The mechanism of starch complexation with edible oils in the presence of a catalyst (K_2_CO_3_) during the extrusion process remains not fully understood. However, analysis of the FTIR spectra revealed the appearance of an intense band at 1741–1743 cm^−1^, indicating the formation of ester bonds (Table 3). The formation of such bonds is evidenced by changes in the intensity ratio of the 1231 cm^−1^ band, which is typically attributed to cross-linking, i.e., the formation of cross-links between the aligned amylose chains in gelatinized starch. In the context of viscosity change analysis (Section 2.2), the occurrence of cross-links influences changes in gel viscosity, and two opposing mechanisms are attributed to them. First, cross-linking prevents the loss of amylose released from starch granules and stabilizes the newly formed structure, thereby increasing gel viscosity. Conversely, the increased number of cross-links can cause diffusional resistance [30], hindering water penetration into the newly formed structure, which slows down the granule swelling and reduces viscosity. Regardless of the dominant mechanism, in each case considered, the use of this type of extruded and oil-enriched starch is advantageous for food processing, particularly as a carrier for hydrophobic substances or in the production of films and food packaging, as it significantly facilitates the monitoring of structural changes.

### 2.4. Adsorption Characterization

Table 4 shows the specific surface area (S_BET_), volume, and average diameter of pores present in the starch extrudates before and after oil modification. The S_BET_ values for the control starch extrudate are in the range of 0.02–0.45 m^2^/g. After oil enrichment, these values shift to 0.11–0.19 m^2^/g for rapeseed oil and 0.10–0.29 m^2^/g for sunflower oil. Changes in these results depend not only on the oil type and its concentration but also on the specific methodology used (BET, Langmuir, or BJH).

### 2.5. Stability Assessment by Dynamic Light Scattering and Microelectrophoresis

To assess the potential stability of the starch systems, we examined their liquid dispersions in glycerol using dynamic light scattering and microelectrophoresis. This allowed determining the dependence of the mean diameter and zeta potential on electrophoretic mobility as a function of time, type, and concentration of edible oil.

Table 5 presents estimated particle sizes of the starch extrudates before and after the enrichment with edible oils in liquid glycerol systems.

As indicated in the table title, these measurements are rough because dynamic light scattering is primarily suitable for dilute systems. Nevertheless, it was necessary to perform microelectrophoretic measurements using the same instrument, allowing for the collection of data on the charge accumulated on the granule surface and the electrokinetic potential of the glycerol dispersion. For completeness, static multiple light scattering studies were simultaneously performed on separate batches of the same systems using a Turbiscan (Section 2.6). Since the SLS method was developed specifically for concentrated systems, it provides a more accurate representation of these samples. Taking these data, the changes in starch extrudates dimensions and the stability/instability of the electrokinetic potential, and the curves obtained with DLS and SLS, conclusions regarding the stability of the individual starch systems can be drawn.

Analysis of the electrokinetic potential data for glycerol dispersions for all seven systems studied revealed relatively low values (Table 6). This leads to the general conclusion that electrostatic stabilization is not possible at such low potential values. Despite this, the systems are quite stable as a function of time, i.e., within 2 h of measurements from the moment of completion of dispersion homogenization. According to literature data [27,28], absolute potential values above 20 mV (25 mV) can provide such stability. However, due to the high viscosity of these systems, particularly those containing sunflower oil, the stabilization mechanism likely involves steric effects and/or a combination of both types of stabilization, i.e., electrosteric stabilization.

Taking into account the data presented in Table 5 and Table 6, including the values obtained after establishing equilibrium (2 h), the samples enriched with sunflower oil, including S6, appeared to be the most stable, as evidenced by their low standard deviation values. Consequently, another conclusion can be drawn that this system will be suitable for specific applications.

### 2.6. Stability Assessment by Static Multiple Light Scattering

Turbiscan LAB Expert (Formulaction) was used to verify the effect of two types of oil (rapeseed and sunflower) at three different concentrations on the behavior and stability of starch extrudate dispersion in a glycerol solution. All the samples were analyzed over a 2 h period, with measurements taken every 15 min at 20 °C. As mentioned earlier, this instrument utilizes static light scattering, a technique specifically suitable for studying concentrated, dense, and viscous dispersions.

**Figure 5:**
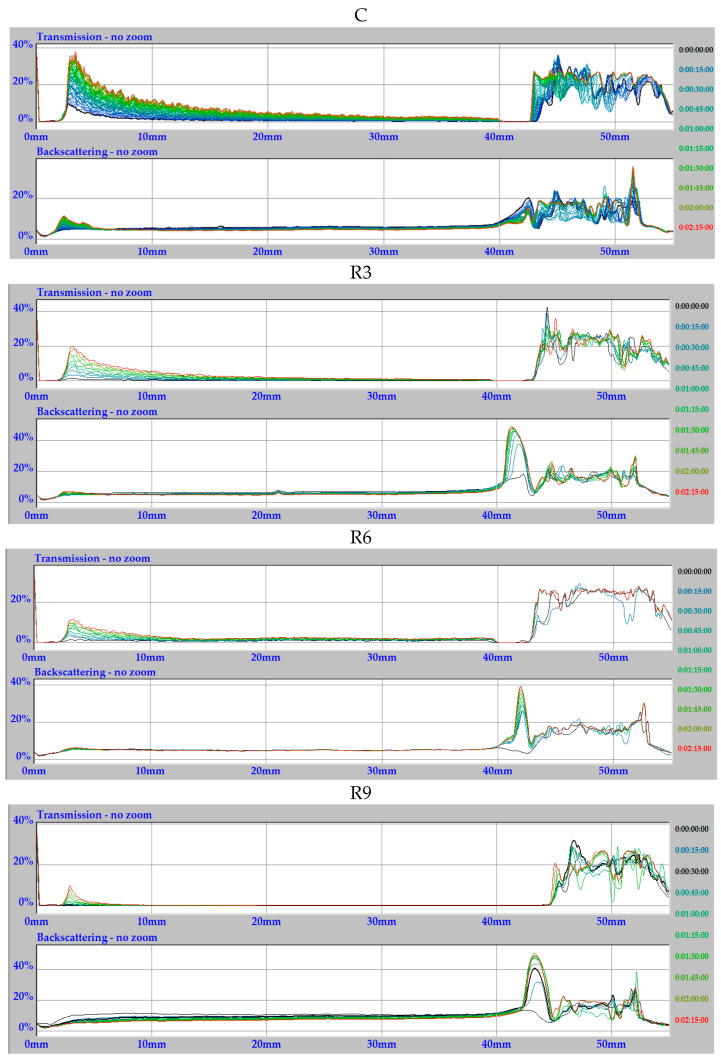
Transmission/backscattering light profiles for dispersions of starch extrudates with and without (C) increasing oil concentrations (R rapeseed oil, 3% R3, 6% R6, and 9% R9) in glycerol systems.

**Figure 6:**
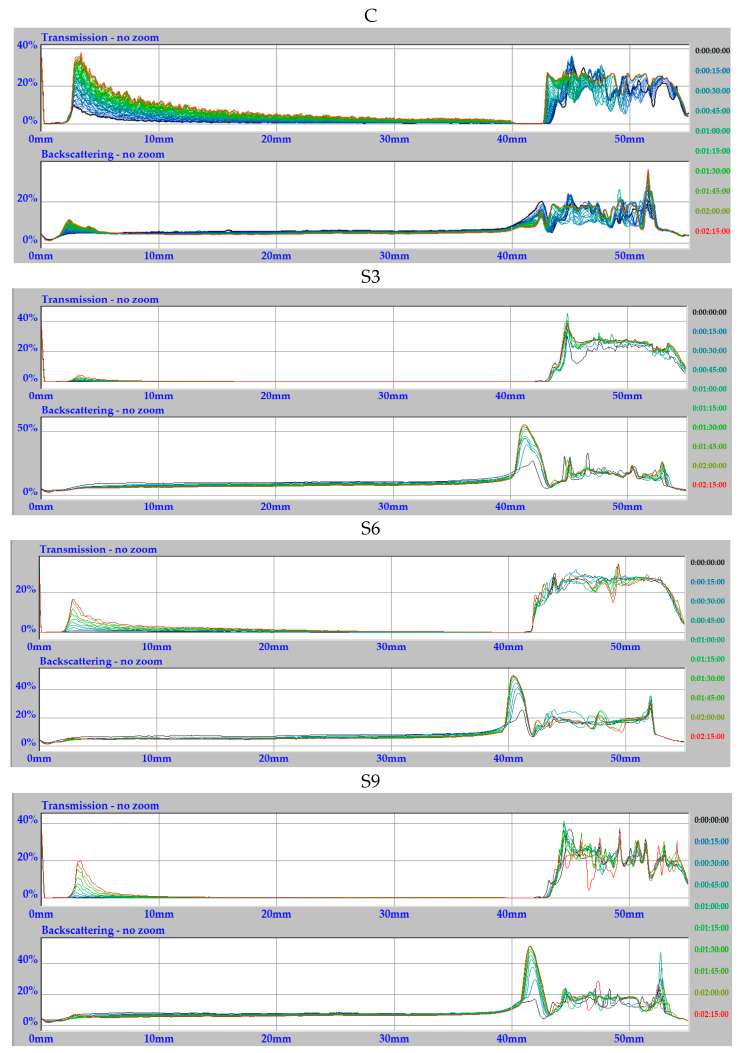
Transmission/backscattering light profiles for dispersions of starch extrudates with and without (C) increasing oil concentrations (S sunflower oil, 3% S3, 6% S6, and 9% S9) in glycerol systems.

To assess how the type and concentration of oil influenced the dispersion, the transmission and backscattering profiles were monitored. These results are illustrated in Figure 5 and Figure 6 for the rapeseed and sunflower oil dispersions, respectively.

The information obtained at this stage was helpful in developing a procedure for creating a stable system for practical applications and further in-depth investigations using other techniques. Figure 5 shows the original relationships obtained directly from the apparatus, where the X-axis represents the cuvette height from 0 mm (bottom of the cuvette) to 55 mm (top of the cuvette). The operating principle requires that the volume of the test solution reach exactly 40 mm, with air above it in the cuvette. Therefore, when analyzing and comparing the profiles obtained for individual samples, we only consider the section on the X axis in the range from 0 mm to 40 mm; changes observed above this value concern air [28,29].

The dispersions tested are stable if the lines of the corresponding scans (for example, taken every 15 min as in this case) overlap. The profiles recorded for each dispersion were analyzed and expressed either (i) as the average Δ% T or BS variation measured at the early zone of the profiles (0–10 mm) corresponding to the sedimentation zone; (ii) the middle zone of the profiles (10–30 mm) as a function of time, which is related to the variation in starch extrudate size; or (iii) the end of the profiles (30–40 mm), which corresponds to creaming/clarification zone. Fluctuations at the bottom (and/or top) of the cuvette and an elongated band may indicate the initiation of the destabilization process. As can be seen in Figure 5, the curve for the unmodified starch extrudate dispersion differs significantly from those of the three samples enriched with rapeseed oil at concentrations of 3%, 6%, and 9% (Figure 5). For the control starch, the light transmission ranges from a few to nearly 40%. The lines are clearly separated, particularly at the bottom of the cuvettes, indicating significant instability. Changes in both light transmission and backscattering observed throughout the dispersion volume during 2 h can suggest slight sedimentation of the control starch.

In turn, the samples with rapeseed oil show significantly greater stability, which was maintained throughout the 2 h measurement period (Figure 5). Practically over the entire volume of the dispersion, i.e., from 0 to 40 mm, the individual scan profiles overlap almost perfectly. This alignment indicates consistent light scattering behavior over time, confirming the absence of destabilization processes. This can also suggest that the addition of oil did not cause any reactions associated with precipitation or fusion of the control starch.

Minimal changes are visible only at the bottom of the cuvette in the transmission light profiles, in the form of small peaks (max. 20% for R3 in descending order R3 > R6 ≥ R9). This may indicate a slight shift in the position of the heavier or more mobile pieces of extruded starch (Figure 5). An interesting dependence on the concentration of the oil used for modification can also be observed. The system with 3% rapeseed oil appears the least stable over the two-hour measurement period. For the 6% and 9% samples, these slight instabilities visible as peaks at the bottom of the cuvette are in fact comparable, despite appearing different at first glance due to differences in the software’s axis scaling. In both cases, the light transmission changes by about 10%.

Based on the light transmission profiles alone, the sample with 9% rapeseed oil appears slightly more stable. Backscattering light profiles can further support these preliminary conclusions. For all the samples with oil content ranging from 3% to 9%, backscattering light profiles were obtained close to 0%. However, a thorough analysis of the backscattering light as a function of time and cuvette height indicates that the highest stability is achieved with 6% oil content. Only in this case, all the scans have a nearly identical progression, and at first glance, they appear to be a single scan. Individual scans are difficult to distinguish, even with a very bright, expressive color scheme available in the camera software (Turbiscan 1.13 Expert), as indicated in the time legend to the right of each image.

The next figure (Figure 6) shows the profiles obtained for analogous dispersions of the starch extrudates enriched with sunflower oil at the same concentrations. For comparison, the relationship for the unmodified starch dispersion is presented at the top (Figure 6). Similarly to the rapeseed oil dispersion, these relationships also confirm the stabilizing effect of the oil. The particles of the oil-enriched starch extrudates exhibit a stronger hydrophobic character and a lower tendency to aggregate, resulting in improved stability over time. This is visible in both the transmitted and backscattering light profiles (Figure 6), where the curves remain largely consistent throughout the analysis.

A noticeable difference between the rapeseed oil (Figure 5) and sunflower oil (Figure 6) dispersions is that the peaks at the bottom of the cuvette obtained for transmission light profiles are at a slightly lower level than previously (slightly over 0% for S3 and S6), confirming the greater stability of the sunflower oil systems. Furthermore, the maximum value of 20% was achieved for sample S9 (i.e., for the highest oil concentration), whereas in rapeseed systems, the highest transmission occurred in sample R3 (the lowest concentration). This trend correlates well with the different viscosity vs. temperature relationships for these systems, discussed in detail in Section 2.2.

Overall, the data for both oils indicate that their presence significantly improves dispersion stability compared to the oil-free control. The dominant factor is likely the fluctuating viscosity of the dispersions as a function of oil concentration, confirmed also by subsequent turbidity studies discussed in Section 2.7 and the stabilizing effect of oil enrichment of the starch extrudates.

### 2.7. Nephelometric Analysis

Turbidity measurements were performed for dispersions of the starch extrudates modified with two types of oil, each at three concentrations. Measurements were taken as a function of time, i.e., over a 2 h period at 15 min intervals, counting from the end of the homogenization process. Similar time ranges, used in most methods, were selected to enable comparative interpretation of the results presented in Table 7, with turbidity expressed in NTU (nephelometric turbidity units).

In both cases, after the enrichment with rapeseed and sunflower oil, the starch extrudate dispersions exhibited higher turbidity values, sometimes even several times higher than the dispersions before modification (401 NTU–696 NTU). The turbidity values for the starch extrudate dispersions modified with rapeseed oil ranged from 545 NTU to 3408 NTU, while for the corresponding dispersions with sunflower oil, they were from 703 NTU to 2322 NTU, respectively. Although these values decreased over time for all the tested dispersions, no clear correlation was found as a function of oil concentration. The lowest turbidity values and the smallest changes over time were observed for samples R6 and S6. This is consistent with the results from the other techniques and indirectly confirms the highest stability of these samples compared to the others.

## 3. Materials and Methods

### 3.1. Materials

The materials used for the tests were extrudates obtained from potato starch (produced by Potato Industry Plant “Lublin”, Lublin, Poland) and sunflower or rapeseed oil (produced by Fat Plant “Kruszwica”, Kruszwica, Poland). Potato starch was combined with edible oil in the proportions specified in Table 8 and thoroughly mixed for 10 min at 150 rpm. Following this, a constant amount of 3 g of catalyst was added to the homogenized mixture. The mixture was then standardized to a moisture content of 22%.

Extrusion was carried out on the Evolum EV-25 (Clextral SAS, Firminy, France) extruder with an output of 7 kg/h, maintaining constant parameters: moisture (22%), screw rotation speed (200 rpm), and nozzle diameter (3.5 mm) (Table 8). The samples obtained for the tests required special preparation, i.e., grinding and sieving through a sieve with a mesh size of 0.63 μm.

### 3.2. Methods

#### 3.2.1. Microscopic Imaging

A Nikon MA 200 microscope (Nikon Solutions Co., Ltd., Tokyo, Japan) was used for the measurements for all the tested samples of control starch and oil-enriched starch extrudates. This device is indispensable for examining large or irregularly shaped samples. The microscope is designed for fluorescence, transmitted light, and confocal microscopy with the following specifications: Nomarski contrast, magnification from 50× to 2000×, epi and dia functions, equipped with a polarizer and 5-megapixel CCD camera.

Optical microscopy is still a fundamental tool in the study of specimens used in biotechnology, medicine, and the food industry. To better visualize preparations and their components, various staining methods are often used, especially for typical biological preparations. No additional staining was used in our studies. Initial visualizations were performed using a conventional light microscope, followed by analysis with a confocal attachment. The technique is based on the phenomenon of fluorescence (the emission of visible light upon exposure to ultraviolet, blue, or green radiation). The natural fluorescence of probes allows for improved visualization of specific structures/processes upon excitation with light of a defined wavelength.

Confocal microscopy is a more advanced, modern variety of fluorescence microscopy that utilizes a laser as the light source. The basic idea is to eliminate images originating from outside the focal plane, which allows for obtaining an image with high sharpness and significantly reduced background noise. A laser beam of a specific wavelength is focused on a single point of the observed object, and the light reflected from this point passes through the optical system and reaches a detector. A three-dimensional image is created point-by-point, thanks to the step-by-step movement of the beam.

#### 3.2.2. Viscosity Measurements

To determine the viscosity curve over a temperature–time gradient, 5% dispersions (aqueous, glycerol) of ground extrudates were prepared. A Rheotest-2 device (VEB MLW Prüfgeräte-Werk Medingen, Medingen, Germany) with an S/S1 cylinder set was used for the determination. The viscosity curve was obtained at a shear rate of 13.5 s^−1^ and a rotational speed of 5 min^−1^. The viscosity value was recorded every 5 min, corresponding to a temperature increase/decrease of 5 °C. Measurements were taken during heating from 20 °C to ~100 °C and cooling to 20 °C. The dynamic viscosity ƞ was determined using the following equation:

$$ƞ=\;\frac{\tau_{r}}{D_{r}}$$

where

ƞ—dynamic viscosity (mPa·s).

$\tau_{r}$—shear stress (Pa).

$D_{r}$—shear rate (1/s).

#### 3.2.3. FTIR

The infrared spectra of starch extrudates, both with and without the addition of edible oils, were recorded using an FTIR spectrometer (model ALPHA II, Bruker Optics Inc., Billerica, MA, USA) employing the Attenuated Total Reflectance (ATR) technique. The ATR method allows for direct sample analysis with minimal preparation. The sample was placed on top of a diamond crystal. The FTIR spectrometer was computer-controlled via the OPUS 8.5 SP1 software, which enabled graphical and mathematical processing of the recorded spectra in the range of 400 cm^−1^ to 4000 cm^−1^.

#### 3.2.4. Adsorption Measurements

The internal structure of control and oil-modified starch extrudates was studied using nitrogen adsorption isotherms. The isotherms were measured using an ASAP 2420 apparatus (Micromeritics Inc., Norcross, GA, USA) at liquid nitrogen temperature (−195.85 °C) and at relative pressures ranging from 0.004 to 0.997 p/p_0_. Measurements were performed on samples weighing from 3 g to 4 g, depending on the specific surface area. Before measurements, the samples were dried for 24 h in a vacuum at 40 °C, automatically desorbed, and flushed with pure helium. Pore volume was calculated using computer software (ASAP 2420 V2.09) included with the apparatus. The specific surface area of the tested starch extrudates was assessed using nitrogen sorption isotherms using the BET method [31]. Pore characteristic was determined according to the BJH method [32,33].

#### 3.2.5. Static Multiple Light Scattering

For the measurements described in Section 3.2.5, Section 3.2.6 and Section 3.2.7, the control sample and extrudates with different additions of edible oils (sunflower and rapeseed) required special preparation. Therefore, starch dispersions in glycerol were prepared at a concentration of 0.5% (g/mL) and mixed for 5 min at a homogenizer speed of 5000 rpm. Immediately after preparation, the samples were divided into several parts and analyzed simultaneously using a few selected techniques described below.

Turbiscan LAB Expert (Formulaction, Toulouse, France) was used to verify the effect of two types of oil (sunflower or rapeseed) at three different concentrations on the behavior and stability of starch extrudate dispersion in a glycerol solution. This optical device allows for the characterization of the actual dispersion state using static multiple light scattering (SLS or SMLS). SLS is the most suitable method for directly characterizing concentrated liquids or highly viscous systems in their native state, without diluting or disrupting the initial form. Consequently, it overcomes the limitations associated with other optical methods, such as laser diffraction or dynamic light scattering, in which concentrated dispersions must be diluted and mixed, changing the state of the system under the influence of mechanical stress. The Turbiscan is equipped with a light-emitting diode with a focused near-infrared band (λ = 880 nm) and two synchronized transmittance and backscatter sensors. All samples were analyzed for 2 h every 15 min at 20 °C ± 1 °C.

#### 3.2.6. Dynamic Light Scattering and Electrophoretic Measurements

The electrokinetic properties of the systems at natural pH were studied by measuring the mean diameter and multimodal particle size distribution. Electrophoretic mobility values for starch systems without and after enrichment with edible oil at three different concentrations were measured using dynamic light scattering and microelectrophoresis (Zetasizer Nano-ZS, Malvern Instruments, Malvern, UK) as a function of time, i.e., after 15, 30, 60, and 120 min, starting from the preparation of the systems. The instrument software converts the obtained values to zeta potential (ζ) according to the Smoluchowski equation. Stability studies using DLS and microelectrophoresis, similarly to SLS, were conducted at room temperature, 20 °C ± 1 °C. Other critical parameters for DLS and electrophoretic measurements are as follows: temperature control (5–75 °C), electric field strength (0–3.2 kV/m), mobility range (10^−9^ to 10^−7^ m^2^/V·s), zeta potential range (±150 mV), and apparatus equipment (red laser, Au electrode, specific cuvette types: disposable/glass).

Zeta potential, one of the most important stability parameters of colloidal systems, is the electrical potential in the double interfacial layer at the slip plane. Zeta potential is not a property of the particle, but of the system; it is the potential difference between the dispersion medium and the stationary fluid layer attached to the dispersed particle. It depends on the type of molecules, the dispersion medium, and the ions present. Consequently, changes to the medium, such as pH, type, and concentration of ions, will simultaneously affect the zeta potential. Knowledge of this parameter allows for understanding properties of dispersion systems and facilitates the interpretation of results obtained from complementary analytical techniques.

#### 3.2.7. Nephelometric Measurements

Nephelometric measurements should complement viscosity measurements, stability, and electrophoresis studies. Therefore, in the next stage of the study, nephelometric measurements were performed for glycerol dispersions of starch extrudates analogously as a function of time, up to 2 h. A HI88713 turbidimeter (HANNA Instruments, Woonsocket, RI, USA) was used for nephelometric studies. This is a high-accuracy laboratory turbidimeter (range 0–4000 NTU ± 2%, nephelometric turbidity unit) with the ISO 7027 requirements [34] for turbidimetric measurements. The instrument is equipped with a red LED source and a photosensitive silicon sensor as the light detector. Similarly to the other methods, all the measurements were performed at room temperature, 20 ± 1 °C.

The nephelometric method is a classical analytical method based on measuring the intensity of light scattered (or reflected) by particles. Intensity measurements were made at a 90° angle to the light beam incident on the system under study, rather than in transmitted light, as in the spectrometric method. The behavior of particles of different sizes and different optical properties under the influence of light rays is described by the Mie theory. For some materials with particle sizes smaller than the wavelength of light, it is possible to apply a simplified Mie theory, the so-called Rayleigh–Gans–Debye theory. All particles smaller than 1/10 the wavelength of the incident light beam behave according to Rayleigh theory, i.e., they scatter light isotropically. This allows for the introduction of a dependence of the scattered light intensity by spherical and colorless particles on the incident light intensity. Turbidity is expressed in NTU (nephelometric turbidity units). Its changes allow monitoring of processes occurring in starch dispersions.

#### 3.2.8. Statistical Analysis

The obtained experimental results were averaged (x), and the standard deviation (σ) was calculated. Data points falling outside the range (x − σ; x + σ) were rejected. For the methodologies described in Section 3.2.5 and Section 3.2.6, the results were subjected to statistical analysis in the PQStat program (version 1.8.6, PQStat Software, Poznań, Poland). One-way analysis of variance (ANOVA) was employed to compare the data, followed by Tukey’s post hoc test to determine the significance of differences between the group mean values. Statistical hypotheses were verified at the significance level of *p* < 0.05.

## 4. Conclusions and Future Perspectives

Enriching starch extrudates with two selected types of oil (rapeseed and sunflower) significantly altered their physicochemical and functional properties. These changes in terms of structure (porosity and adsorption capacity), viscosity, chemical nature, and surface charge were closely dependent on the type of oil used and its concentration. For the oil-enriched starch extrudates, the obtained peak viscosity values were lower than those of the control sample without oil. Notably, extrudates with 6% and 9% rapeseed oil additions were characterized by high viscosity stability (close to constant) during heating. Among the samples enriched with sunflower oil, the sample with 6% oil showed a peak viscosity curve and values similar to its counterparts with rapeseed oil. While porosity decreased slightly with sunflower oil and more substantially with rapeseed oil, both additions resulted in a promising dual hydro/lipophilic character of the starch extrudates.

The FTIR spectra of the samples showed characteristic peaks for the starch material and oils. Specifically, the appearance of a peak corresponding to the carbonyl group of the esters and a peak indicating crystallinity can prove at least partial complexation of oils with starch during the extrusion process. These peaks also point to a slight increase in the structural ordering of the samples. Furthermore, changes in the electrokinetic potential, turbidity parameters, and electrosteric stability of individual starch systems in glycerol also correlated quite well with changes in the internal parameters of the starch. This suggests that these methods are effective tools for assessing the enrichment processes. Based on all the techniques used, the samples enriched with 6% oil appear to be the most promising. In particular, the sample containing 6% sunflower oil stands out due to its higher structural arrangement and stability of its glycerol dispersion.

We hope that the obtained physicochemical characteristics of the analyzed starch-based systems will be a valuable guideline in designing the qualitative and quantitative composition of mixtures (starch and edible oils) intended for films and/or food coatings, dedicated to food with different properties and stored in different environmental conditions.

## Figures and Tables

**Table 1:** Microscopic images of starch extrudates with and without (C) increasing rapeseed oil concentrations (3% R3, 6% R6, and 9% R9).

Code	Optical Microscope	Confocal 408 nm
C	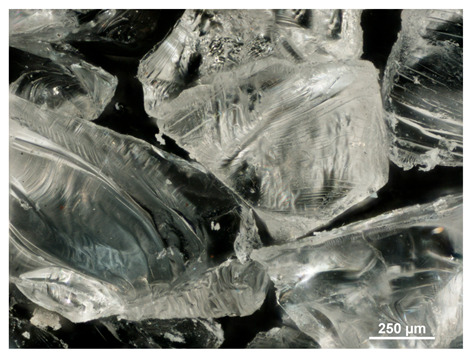	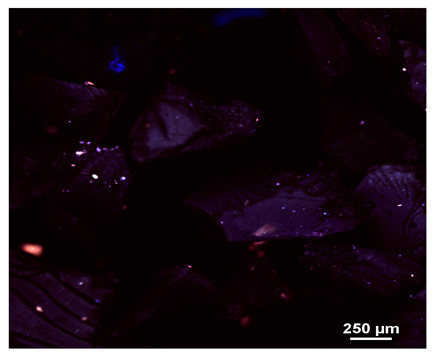
	With oil	
R3	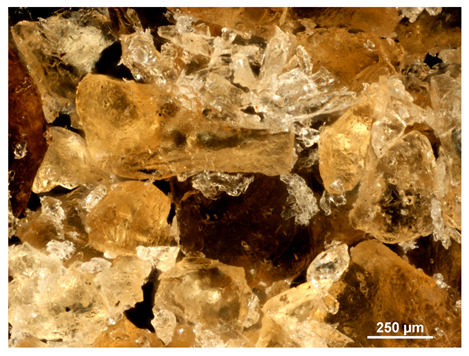	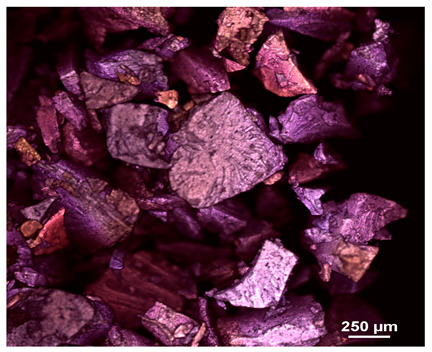

R6	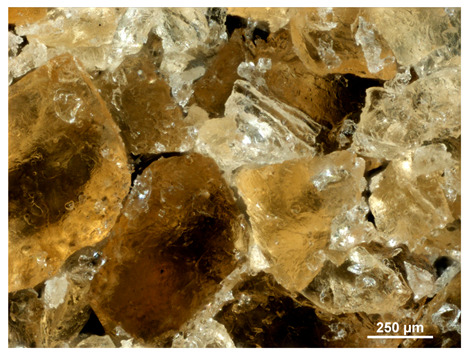	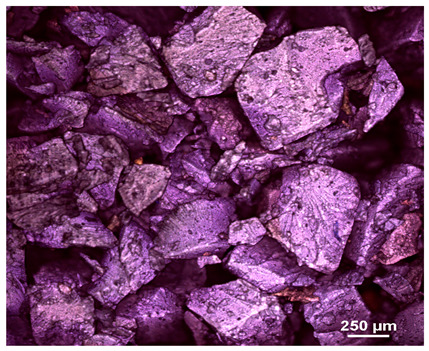

R9	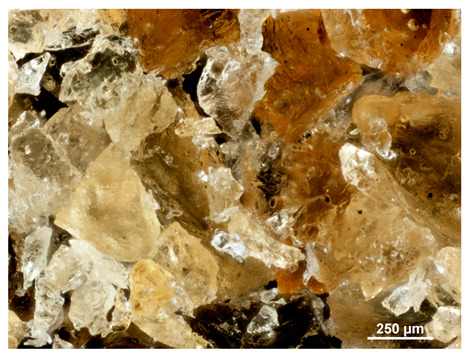	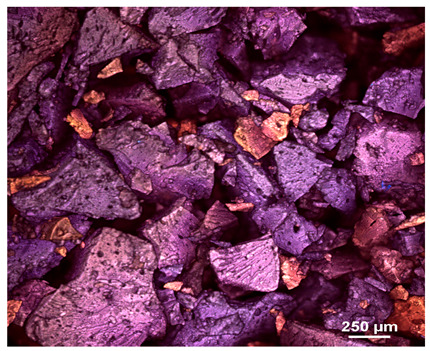

**Table 2:** Microscopic images of starch extrudates with and without (C) increasing sunflower oil concentrations (3% S3, 6% S6, and 9% S9).

Code	Optical Microscope	Confocal 408 nm
C	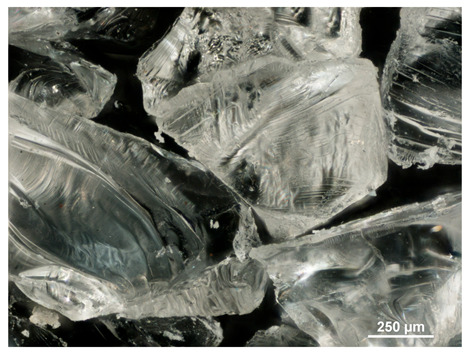	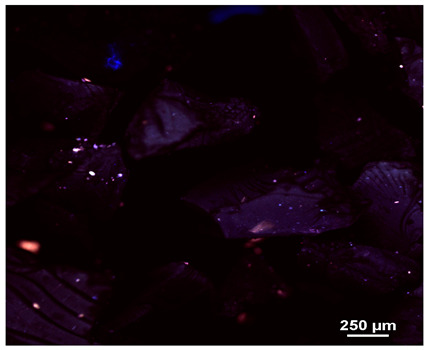
	With oil	
S3	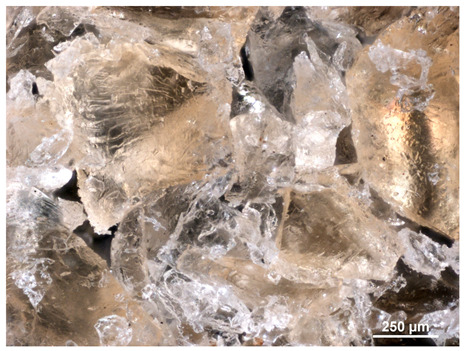	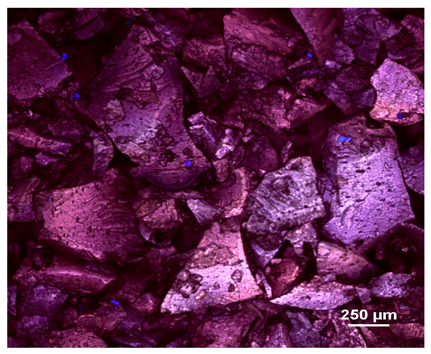

S6	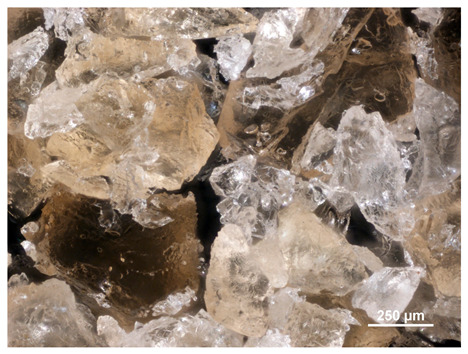	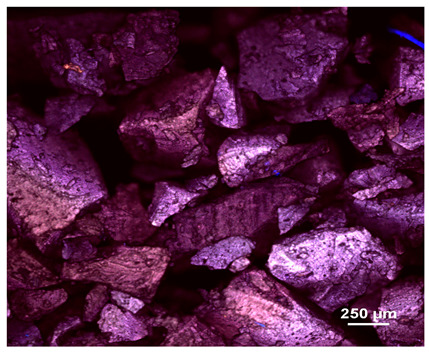

S9	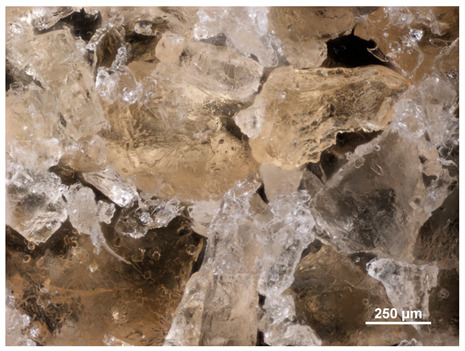	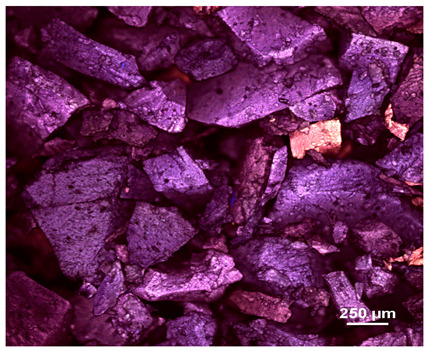

**Table 3:** Summary of major infrared bands in starch extrudates with and without (C) increasing oil concentrations (R rapeseed oil, S sunflower oil, 3% R3 or S3, 6% R6 or S6, and 9% R9 or S9).

Range *v* (cm^−1^)	2850–2950 cm^−1^		1450–1470 cm^−1^		1735–1750 cm^−1^		1100–1250 cm^−1^		Changes in Crystallinity
Type of Vibration	C–H *		CH_2_/CH_3_ **		C=O ***		C–O–C ****		1047/1022
	*v* (cm^−1^)	Intensity Ratio	*v* (cm^−1^)	Intensity Ratio	*v* (cm^−1^)	Intensity Ratio	*v* (cm^−1^)	Intensity Ratio	
C	-	-	-	-	-	-	1142	0.070	0.6795
	2921	0.038							
R3	2856	0.024	-	-	1742	0.020	1146	0.044	0.7168
	2922	0.033							
R6	2855	0.035	1452	0.027	1743	0.036	1148	0.054	0.7346
	2923	0.054							
R9	2856	0.034	1452	0.025	1743	0.034	1149	0.050	0.7543
	2923	0.055							
S3	2857	0.032	1146	0.058	1741	0.024	1146	0.058	0.7020
	2921	0.043							
S6	2856	0.053	1451	0.046	1742	0.050	1148	0.095	0.7314
	2922	0.082							
S9	2856	0.053	1451	0.046	1742	0.050	1148	0.095	0.7175
	2922	0.082							

*v*—wavenumber; * C–H stretching, typical bands for –CH_2_– and –CH_3_ groups in aliphatic chains (often associated with fatty acid esters); ** CH_2_/CH_3_—deformation of aliphatic chains in the ester structure; *** C=O stretching, in the presence of the carbonyl group of the ester; **** C–O–C stretching -C–O vibrations in the bond.

**Table 4:** Adsorption data obtained for starch extrudates with and without (C) increasing oil concentrations (R rapeseed oil, S sunflower oil, 3% R3 or S3, 6% R6 or S6, and 9% R9 or S9) using LTNA method (low-temperature nitrogen adsorption).

Code	C	R3	R6	R9	S3	S6	S9
Specific surface area (m^2^/g)							
BET theory	0.3970 ±0.0044	0.0188 ±0.0009	0.0174 ±0.0017	0.0174 ±0.0004	0.0279 ±0.0001	0.0276 ±0.0005	0.0276 ±0.0002
Langmuir theory	0.4540 ±0.0035	0.0151 ±0.0001	0.0171 ±0.0021	0.0172 ±0.0064	0.0241 ±0.0013	0.0285 ±0.0019	0.0279 ±0.0052
BJH theory for adsorption *	0.0200 ±0.0057	0.0140 ±0.0001	0.0110 ±0.0010	0.0135 ±0.0035	0.0165 ±0.0007	0.0190 ±0.0026	0.0155 ±0.0064
BJH theory for desorption *	0.0204 ±0.0049	0.0114 ±0.0025	0.0116 ±0.0013	0.0127 ±0.0016	0.0103 ±0.0021	0.0227 ±0.0011	0.0168 ±0.0076
Pore volume (10^−4^ m^3^/g)							
BJH theory for adsorption *	1.805	0.895	0.980	1.455	1.400	1.230	1.060
BJH theory for desorption *	1.750	0.735	0.940	0.855	0.630	1.220	1.055
Pore size (nm)							
Adsorption average pore width	18.653	19.072	22.622	25.186	12.363	17.764	15.352
BJH theory for adsorption **	37.689	25.389	34.677	41.150	34.006	25.654	28.207
BJH theory for desorption **	35.381	26.516	32.398	26.612	24.119	21.610	27.101

BET—specific surface area calculated from the Brunauer–Emmett–Teller theory; BJH—porosity determined by the Barrett–Joyner–Halenda method; * cumulative surface area of pores between 1.7000 and 300.0000 diameter; ** average pore diameter (4 V/A); ±—standard deviation.

**Table 5:** Estimated values of the particle size of starch extrudates with and without (C) increasing oil concentrations (R rapeseed oil, S sunflower oil, 3% R3 or S3, 6% R6 or S6, and 9% R9 or S9) in glycerol systems.

Code	C	R3	R6	R9	S3	S6	S9
Time (min)	Size (Diameter: 10^4^ nm)						
15	10.15 ± 2.07 ^a^	15.43 ± 2.81 ^a^	12.06 ± 2.26 ^a^	9.81 ± 1.60 ^a^	12.18 ± 3.08 ^b^	4.27 ± 1.36 ^a^	7.48 ± 3.69 ^a^
30	27.62 ± 8.70 ^b^	11.27 ± 3.16 ^a^	49.40 ± 2.56 ^b^	41.35 ± 31.80 ^a^	2.71 ± 1.27 ^a^	39.07 ± 35.58 ^a^	42.09 ± 36.16 ^a^
60	14.40 ± 3.46 ^a^	18.34 ± 7.88 ^a^	8.25 ± 5.35 ^a^	9.41 ± 1.91 ^a^	12.66 ± 1.52 ^b^	19.95 ± 17.44 ^a^	81.34 ± 72.99 ^a^
120	5.56 ± 1.40 ^a^	13.91 ± 4.08 ^a^	11.32 ± 3.21 ^a^	7.91 ± 1.38 ^a^	7.03 ± 1.18 ^b^	4.45 ± 1.09 ^a^	29.28 ± 5.84 ^a^

The same letters in columns indicate values that are not significantly different at *p* = 0.05; ±—standard deviation.

**Table 6:** Zeta potential values of starch extrudates with and without (C) increasing oil concentrations (R rapeseed oil, S sunflower oil, 3% R3 or S3, 6% R6 or S6, and 9% R9 or S9) in glycerol systems.

Code	C	R3	R6	R9	S3	S6	S9
Time (min)		Zeta Potential (mV)					
16	0.03 ± 0.09 ^b^	−0.10 ± 0.17 ^a^	−0.23 ± 0.18 ^a^	−0.15 ± 0.03 ^a^	−0.23 ± 0.18 ^a^	−0.123 ± 0.08 ^a^	0.070 ± 0.07 ^a^
30	−0.35 ± 0.11 ^ab^	−0.40 ± 0.17 ^a^	−0.23 ± 0.08 ^a^	−0.14 ± 0.06 ^a^	−0.23 ± 0.08 ^a^	−0.05 ± 0.13 ^a^	−0.09 ± 0.08 ^a^
60	−0.05 ± 0.04 ^b^	−0.20 ± 0.06 ^a^	−029 ± 0.08 ^a^	0.16 ± 0.06 ^b^	0.10 ± 0.34 ^a^	0.10 ± 0.34 ^a^	−0.19 ± 0.07 ^a^
120	−0.64 ± 0.32 ^a^	−0.15 ± 0.06 ^a^	−0.08 ± 0.10 ^a^	−0.12 ± 0.10 ^a^	−0.15 ± 0.11 ^a^	−0.15 ± 0.11 ^a^	−0.33 ± 0.30 ^a^

The same letters in columns indicate values that are not significantly different at *p* = 0.05; ±—standard deviation.

**Table 7:** Turbidity measured for dispersions of starch extrudates with and without (C) increasing oil concentrations (R rapeseed oil, S sunflower oil, 3% R3 or S3, 6% R6 or S6, and 9% R9 or S9) in glycerol systems as a function of time.

Code	C	R3	R6	R9	S3	S6	S9
Time (min)	Turbidity of Starch Extrudate Dispersion (NTU)						
0	696	3408	975	2390	2322	1423	1530
15	668	3211	863	2343	2056	1098	1199
30	600	3006	767	2186	1804	1028	1100
45	541	2834	701	1863	1695	990	1033
60	541	2664	658	1736	1598	916	937
75	495	2542	626	1614	1517	876	868
90	449	2415	598	1502	1389	834	809
105	425	2236	567	1363	1296	796	748
120	401	2130	545	1267	1220	771	703

**Table 8:** The qualitative and quantitative characteristics of starch-oil composition used for extrusion (%).

Code	C	R3	R6	R9	S3	S6	S9
Weight composition (g/100 g)							
Potato starch	100	94	91	88	94	91	88
Catalyst K_2_CO_3_	0	3	3	3	3	3	3
Rapeseed oil	0	3	6	9	-	-	-
Sunflower oil	0	-	-	-	3	6	9
Characteristics of extrusion conditions							
Temperature profile	100/100/100/75/75/60 (°C)						
Screw speed	200 (rpm)						
Nozzle diameter	3.5 (mm)						
Moisture	22 (%)						

## Data Availability

The original contributions presented in this study are included in the article material. Further inquiries can be directed to the corresponding author(s).
